# Use of health care services and pharmaceutical agents in coeliac disease: a prospective nationwide study

**DOI:** 10.1186/1471-230X-12-136

**Published:** 2012-09-27

**Authors:** Anniina Ukkola, Kalle Kurppa, Pekka Collin, Heini Huhtala, Leena Forma, Leila Kekkonen, Markku Mäki, Katri Kaukinen

**Affiliations:** 1 School of Medicine, FinnMedi3, University of Tampere, Biokatu 10, FIN-33014, Tampere, Finland; 2 Paediatric Research Centre, University of Tampere and Tampere University Hospital, Tampere, Finland; 3 Department of Gastroenterology and Alimentary Tract Surgery, Tampere University Hospital, Tampere, Finland; 4 School of Health Sciences, University of Tampere, Tampere, Finland; 5 Finnish Coeliac Society, Tampere, Finland

**Keywords:** Coeliac disease, Gluten-free diet, Burden of illness, Health care service use, Sickness absence

## Abstract

**Background:**

Approximately 1% of the population suffer from coeliac disease. However, the disease is heavily underdiagnosed. Unexplained symptoms may lead to incremented medical consultations and productivity losses. The aim here was to estimate the possible concealed burden of untreated coeliac disease and the effects of a gluten-free diet.

**Methods:**

A nationwide cohort of 700 newly detected adult coeliac patients were prospectively evaluated. Health care service use and sickness absence from work during the year before diagnosis were compared with those in the general population; the data obtained from an earlier study. Additionally, the effect of one year on dietary treatment on the aforementioned parameters and on consumption of pharmaceutical agents was assessed.

**Results:**

Untreated coeliac patients used primary health care services more frequently than the general population. On a gluten-free diet, visits to primary care decreased significantly from a mean 3.6 to 2.3. The consumption of medicines for dyspepsia (from 3.7 to 2.4 pills/month) and painkillers (6.8-5.5 pills/month) and the number of antibiotic courses (0.6-0.5 prescriptions/year) was reduced. There were no changes in hospitalizations, outpatient visits to secondary and tertiary care, use of other medical services, or sickness absence, but the consumption of nutritional supplements increased on treatment.

**Conclusions:**

Coeliac disease was associated with excessive health care service use and consumption of drugs before diagnosis. Dietary treatment resulted in a diminished burden to the health care system and lower use of on-demand medicines and antibiotic treatment. The results support an augmented diagnostic approach to reduce underdiagnosis of coeliac disease.

**Trial registration:**

ClinicalTrials.gov NCT01145287

## Background

Untreated coeliac disease may cause a significant burden to the health care system. The disease is one of the commonest chronic gastrointestinal disorders, with a prevalence of up to 2% in the adult population [1], the figure in fact even increasing [2]. The clinical picture is heterogeneous and comprises mild or extraintestinal symptoms such as osteoporosis and neurological complaints [3,4]. Up to 90 per cent of coeliac disease patients remain undiagnosed [1,5,6] and may suffer from impaired health and repeatedly seek help for non-specific complaints [7-9]. This may lead to excessive use of health services, for example frequent outpatient visits and expensive medical investigations. Furthermore, physicians or patients themselves may try to treat the unexplained symptoms and poor well-being with a variety of pharmaceutical agents or micronutrients in addition to non-medical treatments. Possible false diagnoses and futile measures might cause a further burden to the health care system. It could also be hypothesized that undetected coeliac disease leads to an increased number of days of absence from work. As treatment with a gluten-free diet usually results in alleviation of symptoms it could thus also diminish the burden related to the disease. Remarkably, the mean diagnostic delay in coeliac disease is between 4 and 13 years [8-12], indicating that the burden caused by undiagnosed disease might be long-standing. Now that accurate serological screening tests are available for screening for coeliac disease, an active screening policy would shorten this period of latency and reduce the burden related to undetected disease. The data thus far available on the use of health care services, consumption of symptom-targeted medication and sickness absence among undiagnosed and treated coeliac disease patients are limited.

In this country, an increasing number of coeliac disease patients are diagnosed in primary health care [13]. Due to intensified case finding and screening, mild or atypical symptoms dominate the clinical picture and the detection rate is up to 0.7% . We aimed here to estimate prospectively the possible concealed burden of untreated coeliac disease and the effects of a gluten-free diet in a large nationwide cohort of newly detected coeliac disease patients. We also compared the results of health care service use and reported days of absence from work with national data from the general Finnish population during the same period.

## Methods

A nationwide cohort of consecutive newly detected coeliac disease patients were prospectively evaluated. A structured and validated study questionnaire was mailed to all new members joining the Finnish Coeliac Society between February 2007 and May 2008. In Finland, approximately 70% of coeliac disease patients join the Society shortly after being diagnosed. Respondents over 16 years of age with biopsy-proven coeliac disease diagnosed within one year were eligible. A follow-up questionnaire was sent to all respondents after one year. The questionnaires were designed in co-operation with the Finnish Coeliac Society, coeliac disease patients and clinical researchers with expertise in the disease [14], and comprised questions on personal health, clinical presentation and issues related to the diagnosis. Self-reported consumption of on-demand pharmaceutical agents and supplements, both prescribed and over-the-counter agents, was inquired. Patients were asked to report separately for each type of pharmaceutical agent the number of pills per month consumed on average during the years prior to and following the diagnosis. The use of antibiotics was reported as the number of courses during the study periods. The use of health care services, including all-cause visits (inpatient, outpatient, other medical services; the causes of visits were not asked) and also visits related to the diagnosis and follow-up were recorded during one year prior to the diagnosis of coeliac disease and after initiation of a gluten-free diet (patients were asked to fill in the number of each kind of visits). Patients were also asked to report the number of days of sickness absence from work during the same periods. The appropriateness of the questions together with the face and content validity of the tested items were pre-tested by a group of coeliac disease members of the Society as previously described [14]. Test-retest reliability was established using an intraclass correlation coefficient. For the key items measured, the kappa values for test-retest reliability ranged from 0.84 to 1.00 (values above 0.70 being regarded as excellent). For the use of health care services, the kappa values ranged from 0.94 to 1.00, for the consumption of pharmaceutical agents from 0.90 to 1.00 and for the number of days of sickness absence 0.99. All data were blindly coded before analyses. The study protocol was approved by the review board of the Finnish Coeliac Society in compliance with all applicable Finnish laws for registered organizations and governing the protection of human subjects. Informed consent was obtained from all study subjects after a full written explanation of the aims of the study, including considerations regarding ethics and data protection and the anonymous deposition of the questionnaires.

Data on self-reported visits to a physician and days of absence from work among the general Finnish population during the same period (2007–2008) were obtained from an annual nationwide postal survey conducted by the National Institute for Health and Welfare since 1978. The surveys are mailed to a random sample of 5000 Finnish adults (15–64 years of age) each year [15,16]. Characteristics of the population controls are shown in Additional file 1. In that study, the participants were asked to report the total number of all-cause medical consultation with a physician including hospitalizations. The number of days of sickness absence was asked similarly as here. In the present study, comparisons on the aforementioned issues between the coeliac group and the general population were limited to study subjects of the same age. The groups were not adjusted for gender.

Statistical analyses were performed using SPSS version 17.0 (SPSS Inc., Chicago, IL, USA). All testing was two-sided and p < 0.05 was considered statistically significant. Chi square test and Fisher’s Exact test were used in cross tabulations, Wilcoxon signed rank test for evaluating changes within groups and Mann–Whitney U test for assessing changes between groups. Results are given as means and 95% confidence intervals (CI).

## Results

The study questionnaires were mailed to 1864 coeliac disease patients, of whom 1062 responded giving a response rate of 57% . There were no differences in age or gender distribution between respondents and non-responders. Altogether 362 individuals were excluded: in 157 cases the diagnosis had been made more than one year previously, 132 were under 16 years of age and 73 did not have a biopsy-proven diagnosis. Thus, 700 adult patients were enrolled (Table 1). Of these, 679 (97% ) also completed the follow-up survey. After one year, 86% of the patients reported adherence to a strict gluten-free diet, only two having continued on a gluten-containing diet.

**Table 1:** Baseline characteristics of the coeliac disease study group

	**All**	**Female**	**Male**
	**n = 700**	**n = 534 (76% )**	**n = 166 (24% )**
Median age (range), years	49 (16–84)	48 (16–84)	53 (16–83)
Clinical presentation, %			
Gastrointestinal symptoms and signs	70	70	70
Extraintestinal symptoms	9	8	12
Detected by screening^*^	21	22	18
Duration of symptoms			
Median (range), years	3 (0–59)	3 (0–51)	2 (0–59)
25-75th percentile	1-7	1-7	1-5
OEGD at diagnosis, %	100	100	100
OEGD prior to diagnosis of CD, % ^*^	21	19	27
Diagnosis was established in, %			
Primary health care	52	55	42
Secondary health care	38	36	44
Tertiary health care	10	9	14

OEGD oesphago-gastroduodenoscopy.

CD coeliac disease.

^*^ Screening performed because of patients belonged to a risk group (first-degree relatives of coeliac disease patients, those with type 1 diabetes mellitus, autoimmune thyroid disease, Sjögren’s syndrome and IgA deficiency).

^†^ Other than the oesphago-gastroduodenoscopy by which the diagnosis of coeliac disease was confirmed.

**Figure 1:**
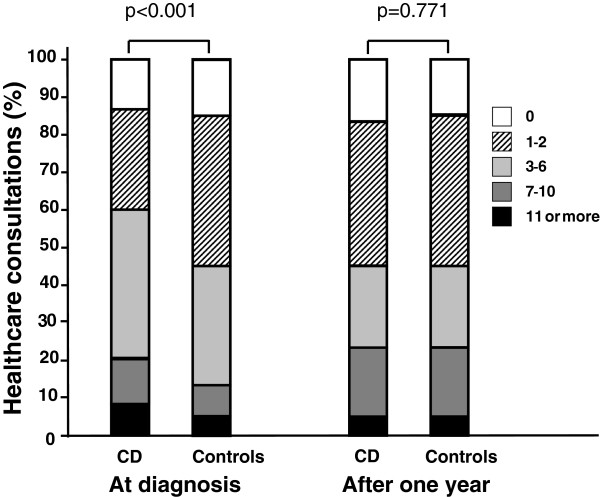
**All-cause outpatient and inpatient consultations with a physician in the year prior to and following the diagnosis of coeliac disease. **The number of consultations is compared with that in the general adult population during the same period and limited to subjects 16–64 years of age (patients n_1_ = 576, n_2_ = 567; controls n_1_ = 3201, n_2_ = 3190).

In the year prior to the diagnosis of coeliac disease, 85% of the patients had consulted a physician in primary health care, 30% in secondary or tertiary health care, 12% had had at lest one admission to a hospital and 60% had used other medical services (consultations with a nurse, a psychologist or a dietician, home nursing care, physiotherapy, laboratory and imaging services). After one year on a gluten-free diet the corresponding proportions were 74% , 26% , 13% and 65% , respectively. In the year prior to the diagnosis, the patients had consulted either a primary care or a hospital physician a mean 4.4 times. Of the respondents, 12% claimed they had not consulted a physician. In the year following the diagnosis while on a gluten-free diet, the corresponding figure was 3.1 and no consultations with a physician was reported by 18% . A significant reduction was observed in use of primary health care services. There were no changes in the number of consultations in secondary or tertiary health care or in admissions to or consumption of other medical services (Table 2). The use of health care services assessed as all-cause consultations with a physician and admissions to a hospital was significantly higher in both genders in the coeliac disease group than in the general population (p < 0.001) (Figure 1). After one year on a gluten-free diet, no such difference was observed. When analysed by age, all age groups (25 to 34, 35 to 44, 45 to 44 and 55 to 64 years old) except the youngest age group (16 to 24 years old) had significantly more consultations than the population controls at baseline, p = 0.07, p < 0.001, p = 0,41 and p = 0.19, respectively. After the follow-up, no differences were noted.

**Table 2:** Changes in the mean number of all-cause medical consultations among coeliac disease patients between the year prior to and after the diagnosis of the disease

	**All**	**Female**	**Male**
	**n = 700**	**n = 534**	**n = 166**
Outpatient visits in primary health care			
Year prior to diagnosis	3.6	3.7	3.1
Year after diagnosis on a GFD	2.3	2.5	1.9
Mean change (95% CI)	−1.2 (−1.5 to −0.9)	−1.3 (−1.6 to −0.9)	−1.2 (−1.7 to −0.7)
P value	<0.001	<0.001	<0.001
Outpatient visits in secondary and tertiary health care			
Year prior to diagnosis	0.8	0.8	0.8
Year after diagnosis on a GFD	0.8	0.8	0.9
Mean change (95% CI)	0.0 (−0.2 to 0.1)	0.0 (−0.2 to 0.2)	0.0 (−0.3 to 0.4)
P value	0.664	0.630	0.906
Admissions to hospital			
Year prior to diagnosis	0.2	0.2	0.2
Year after diagnosis on a GFD	0.2	0.2	0.2
Mean change (95% CI)	0.0 (−0.03 to 0.1)	0.0 (−0.1 to 0.1)	0.0 (−0.1 to 0.1)
P value	0.708	0.521	0.724
Other medical services^*^			
Year prior to diagnosis	4.1	4.5	2.7
Year after diagnosis on a GFD	3.6	4.0	2.4
Mean change (95% CI)	−0.5 (−1.0 to 0.1)	−0.5 (−1.2 to 0.2)	−0.3 (−1.2 to 0.7)
P value	0.340	0.245	0.797

GFD gluten-free diet.

CI confidence interval.

^*^ Consultations with a nurse, a psychologist or a dietician, home nursing care, physiotherapy, laboratory and imaging services.

**Figure 2:**
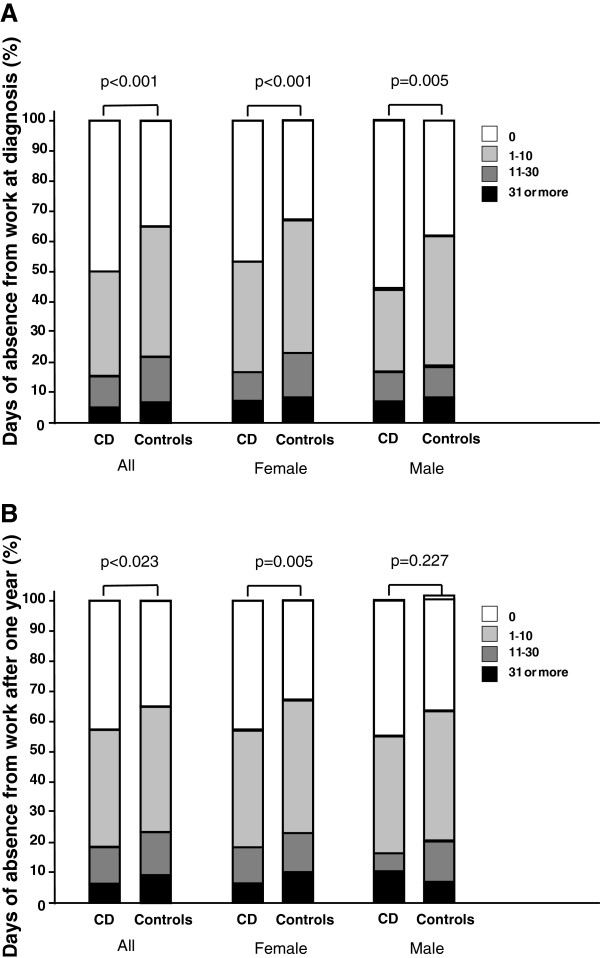
**Days of sickness absence from work in the year prior to (A) and following (B) the diagnosis of coeliac disease. **The number of days of absence is compared with that in the general adult population during the same period and limited to subjects 16–64 years of age and who were not retired (all patients n_1_ = 480, n_2_ = 477; all controls n_1_ = 2949, n_2_ = 2976; female patients n_1_ = 383, n_2_ = 382; female controls n_1_ = 1656, n_2_ = 1693; male patients n_1_ = 97, n_2_ = 95; male controls n_1_ = 1293, n_2_ = 1283).

The reported number of days of sickness absence from work was significantly lower in the coeliac group than in the general population in the year prior to the diagnosis (Figure 2A). Of those at working age and who were not retired (n = 480), 50% stated they had no sickness absence compared to 35% in the general population. After one year, the corresponding figures were 42% and 35% , respectively. In the year following the diagnosis, sickness absences had increased to population level among males but not among females (Figure 2B). When the male group was analysed by age, only the youngest (16 to 24 years old) and the oldest (55 to 64 years old) age groups differed significantly from the controls, p = 0.018 and p = 0.005, respectively. Those 55 to 64 years old had the biggest increase in the number of days of absence, a mean of 5.6 days. Among the youngest age group the mean increase was 1.8 days.

The coeliac disease patients’ consumption of pharmaceutical agents in the year prior to and following the diagnosis is shown in Table 3. There was a significant reduction in all on-demand medicines on treatment. In particular, the use of drugs for dyspepsia decreased significantly in both genders, and that of painkillers and the number of antibiotic prescriptions in females (Table 3). Of the patients, 74% reported having used on-demand medication in the year before the diagnosis and 73% in the year following the diagnosis. For painkillers the percentages of users were 65% and 66% and for medicines for dyspepsia 26% and 18% , respectively. Of the patients, 30% had peen prescribed antibiotics prior to and 26% after the diagnosis.

**Table 3:** Changes in reported consumption of pharmaceutical agents among coeliac disease patients (pills per month on average) between the year prior to and following the diagnosis of coeliac disease

	**All**	**Female**	**Male**
	**n = 700**	**n = 534**	**n = 166**
All on-demand medicines^*^			
Year prior to diagnosis	12.0	12.2	11.4
Year after diagnosis on a GFD	9.3	9.1	10.0
Mean change (95% CI)	−2.7 (−4.3 to −1.2)	−3.1 (−4.9 to −1.4)	−1.4 (−4.8 to 2.0)
P value	<0.001	<0.001	0.011
Painkillers			
Year prior to diagnosis	6.8	7.2	5.6
Year after diagnosis on a GFD	5.5	5.2	6.4
Mean change (95% CI)	−1.3 (−2.6 to −0.1)	−2.0 (−3.4 to −0.5)	0.8 (−2.1 to 3.7)
P value	<0.001	<0.001	0.432
Medicines for dyspepsia			
Year prior to diagnosis	3.7	3.5	4.4
Year after diagnosis on a GFD	2.5	2.4	2.6
Mean change (95% CI)	−1.27 (−2.10 to −0.44)	−1.1 (−2.0 to −1.2)	−1.8 (−3.7 to 0.1)
P value	<0.001	<0.001	0.015
Antibiotic treatment^†^			
Year prior to diagnosis	0.6	0.7	0.4
Year after diagnosis on a GFD	0.5	0.5	0.3
Mean change (95% CI)	−0.1 (−0.2 to −0.1)	−0.2 (−0.3 to −0.1)	−0.1 (−0.3 to 0.1)
P value	0.001	0.001	0.302
Vitamins, micronutrients, herbal products			
Year prior to diagnosis	18.4	20.7	10.8
Year after diagnosis on a GFD	22.6	24.6	16.2
Mean change (95% CI)	4.2 (1.8 to 6.7)	3.9 (0.9 to 6.8)	5.5 (1.8 to 9.1)
P value	<0.001	0.003	0.002

CI confidence interval.

GFD gluten-free diet.

^*^ Painkillers, medicines for dyspepsia, sleeping pills.

^†^ Not reported as pills per month but as number of courses per year.

In contrast to other pharmaceutical agents, the consumption of vitamins, micronutrients and herbal products increased significantly in both genders on a gluten-free diet (Table 3). Males increased their consumption even more than female. While at diagnosis 49% of the patients reported at least occasional use of vitamins, micronutrients or herbal products, the corresponding figure after one year on treatment was 55%.

## Discussion

In this prospective, nationally representative study we found that untreated adults with coeliac disease made more outpatient health care visits than the general population. In addition, implementation of a gluten-free diet resulted in the disappearance of this increased consumption of medical services. The burden of unrecognized coeliac disease was concentrated particularly in primary health care. In parallel to these findings, a significant reduction in the use of on-demand drugs and the number of antibiotic prescriptions was observed while on dietary treatment. To our knowledge, this was the first study to investigate sickness absence and consumption of on-demand medication among coeliac disease patients.

A possible explanation for the increased use of health care services and symptom-targeted medication prior to diagnosis might be related to the presence of diverse symptoms: untreated coeliac disease is known to be associated with various unspecific complaints – e.g. indigestion and heart burn [17], regurgitation [18], migraine [19] and joint pain [20], which may resolve on a gluten-free diet. It has also been suggested that active coeliac disease may be associated with an increased susceptibility to infections [21,22]. Our findings suggest that the diagnosis and subsequent treatment of coeliac disease are able to reduce the burden of disease in the health care system in addition to the alleviated burden experienced by patients [14].

Earlier data on the use of health care services in coeliac disease are limited. Two recent retrospective studies from the USA found that treatment with a gluten-free diet resulted in decreased medical costs due to reduced use of health care services among coeliac disease patients. However, these studies concentrated on direct costs and obtained study participants in high-volume referral centres or administrative claim registers [23,24], which may limit extrapolation of the data to the whole coeliac disease population. It is of note that the main findings - excessive health care service use before the diagnosis of coeliac disease and reduction in the consumption of these services during a gluten-free diet - were in line, despite the difference in settings between these earlier trials and our current prospective nation-wide study. However, in contrast to the results reported by Long and associates [24] we found no difference in the number of hospitalizations between the years prior to and following the diagnosis of coeliac disease. In addition, in the present study expenditure on laboratory services and imaging were not increased prior to diagnosis. Even though direct medical costs were not evaluated in the present study, the results of decreased use of health care services and pharmaceutical agents suggest decrease in costs for the health care system. In addition to the studies mentioned earlier, another recent study showed excess costs of undiagnosed symptomatic coeliac disease patients and highlighted a largely advantageous cost-benefit ratio for a diagnosis of the disease [25]. From an ethical point of view, as the aforementioned studies have shown increased numbers of medical investigations before the diagnosis, it should be noticed how many unnecessary invasive procedures (such as bone biopsy and blood transfusions for unexplained anaemia, colonoscopy for abdominal pain, computed tomography for suspected inexistent neoplasms) could have been saved in these patients if the disease would have been diagnosed earlier.

Nowadays the proportion of coeliac disease patients suffering from severe gastrointestinal symptoms and malabsorption is decreasing and milder symptoms predominate. Consultations on these possibly vague and unspecific symptoms might add to the burden in primary health care, which patients first contact upon any complaints. Additionally, the diagnostics and follow-up of coeliac disease among adults are focused in primary health care in Finland [13], all these aspects possibly explaining the increased use of primary health care services observed in the present study.

Even though the consumption of health care services among coeliac disease patients was reduced to the population level during one year on a gluten-free diet, further studies are needed to establish the long-term impact of dietary treatment. A recent study from Sweden reported that, in spite of a median of 4 years on a gluten-free diet, female coeliac disease patients used more health care services than non-coeliac controls [26]. It was also shown that the majority of complaints were related to gastrointestinal symptoms, mental and behavioural disorders and diseases of the musculoskeletal system. There is further evidence that regardless of a long-term gluten-free diet and histological remission, coeliac disease patients may evince significant symptoms and impaired health-related quality of life [27,28]. A recent study found that impaired quality of life in coeliac disease patients on a gluten-free diet is largely explained by coexistence of irritable bowel syndrome and reflux [29].

It was somewhat surprising that the patients in our study reported no increased sickness absence from work prior to diagnosis. Actually, the number of days of absence was even lower than that among the general population. This would imply that currently the majority of coeliac disease patients present with relatively mild clinical symptoms. However, although untreated coeliac disease is known to be associated with increased anxiety and depression [30], reduced vitality [31] and sleeping disorders [32], we did not here inquire in to the possible decrease in productivity among undetected coeliac disease patients. The reason why male but not female coeliac disease patients increased their number of days of absence from work in the follow-up remains unsolved. Possibly, once given a diagnosis of a chronic disease the patients may have thought to be “validated” or “vindicated” in being off work. Unfortunately, we could not ascertain whether sick leaves were concentrated during the period short after diagnosis or were evenly distributed along the follow-up period.

Even though decrease in the number of antibiotic prescriptions was not big it can be regarded as clinically significant. Two recent studies [33,34] have investigated use of antibiotics and risk of developing Crohn’s disease or ulcerative colitis. They found that subjects with those diseases were more likely to have been prescribed antibiotics before the diagnosis. Shaw and associates [33] speculated that the use of antibiotics could be a predisposing factor, whereas Virta and associates [34] considered that frequent use of antibiotics may trigger the development of Crohn’s disease or be a sign of being prone to infections before the intestinal disease is diagnosed. Our hypothesis about coeliac disease patients is similar to the latter consideration and is supported by the fact that fewer patients had been prescribed antibiotics post than pre-diagnosis. However, whether increased use of antibiotics really is a predisposing factor to coeliac disease or sign of decreased immunity because of undetected coeliac disease is out of the scope of this study and needs further evaluations.

Interestingly, the use of vitamins, micronutrients and herbal products increased significantly after the diagnosis of coeliac disease. It has been reported that about 15-38% of untreated coeliac disease patients suffer from anaemia or nutritional deficiencies [35-37]. Nevertheless, these are usually abolished on a gluten-free diet [35,38], and implementation of specific dietary supplements after the diagnosis is not routinely recommended in current clinical guidelines [39]. Consequently, we believe that in most cases the supplements were not prescribed by a physician but started voluntarily by the patients. This is somewhat worrying as increased use of vitamin and micronutrient supplements in suggested to be related to increased risk for cancer [40]. However, there have been alarming results of dietary shortcomings among those following a gluten-free diet as patients have been reported to consume insufficient amounts of vitamins and micronutrients [41,42]. This might be due to poor nutritional value of commercial gluten-free products or inadequate dietary choices. Thus, on the other hand, dietary supplements may be needed.

The fact that we used self-reported data might be considered a limitation to the study in that it may involve inaccuracies. However, a unique strength of such a setting in a nation-wide study was that by using self-reported data it was possible to explore all aspects of health care use instead of data captured in a single database. Subsequently, we were able to assess not only issues related to direct costs of care but also the indirect burden falling on coeliac disease patients themselves. However, similar methods have previously been used in studies concerning gastrointestinal disorders and a recall period covering the preceding twelve months in self-reported use of health care services and pharmaceutical agents has been shown to be feasible and reliable [43]. Moreover, patients were asked to report issues similarly at baseline and in the follow-up, which makes the changes observed more reliable and test-re-test reliability for the key items was excellent. Likewise, the data on the general population were based on self-reported values asked similarly as in the present study. However, the study questionnaires for the cohorts were different which might have impacted the results. The response rate in this study was relatively low but comparable to those of earlier studies with similar study design [8,44]. A weakness of the study is that because we were unable to ask about co-morbidities it was impossible to assess their possible impact to the results.

## Conclusions

Excessive use of primary health care services and pharmaceutical agents was observed among untreated coeliac disease patients. Treatment with a gluten-free diet resulted in decreased consumption of health care services, on-demand medicines and antibiotic prescriptions. The results imply that unrecognized coeliac disease contributes markedly to the burden on affected individuals and the health care system.

## Abbreviations

OEGD: Oesphago-gastroduodenoscopy; CD: Coeliac disease; GFD: Gluten-free diet; CI: Confidence interval.

## Competing interests

The authors declare that they have no competing interests.

## Authors’ contributions

AU participated in the study design, performed part of the statistical analysis and drafted the manuscript. KKu was involved in the study design and preparing the manuscript. PC participated in the study design and preparing the manuscript. HH performed part of the statistical analysis and critically reviewed the manuscript. LF participated in the interpretation of the data and critically reviewed the manuscript. LK participated in the study design and critically reviewed the manuscript. MM was involved in the study design and critically reviewed the manuscript. KKa participated in the study design and drafting of the manuscript. All authors read and approved the final manuscript.

## Pre-publication history

The pre-publication history for this paper can be accessed here:

http://www.biomedcentral.com/1471-230X/12/136/prepub

## Supplementary Material

Additional file **Characteristics of the population controls**[15,16]. Click here for file
