# Supplementary material for: Use of health care services and pharmaceutical agents in coeliac disease: a prospective nationwide study
Source: BMC Gastroenterol. 2012 Sep 27;12:136. doi: 10.1186/1471-230X-12-136 (PMC3503835; doi:10.1186/1471-230X-12-136)
Supplement: Additional file — Characteristics of the population controls[15,16]. [file 1471-230X-12-136-S1.doc]

Supplementary table. Characteristics of the population controls [15,16]

|  | | All  n=3245 | Female  n=1825 (56%) | Male  n=1420 (44%) |
| --- | --- | --- | --- | --- |
| Age range, years | | 15-64 | 15-64 | 15-64 |
| Marital status, % | |  |  |  |
|  | Married or cohabiting | 66 | 66 | 66 |
|  | Living alone | 34 | 34 | 34 |
| Social index, % | |  |  |  |
|  | I | 9 | 7 | 12 |
|  | II | 38 | 59 | 50 |
|  | III | 35 | 16 | 24 |
|  | Student | 14 | 18 | 17 |
| Incidence of abdominal symptoms or complaints in the past month, % | |  |  |  |
|  | Constipation | 7 | 9 | 4 |
|  | Heartburn | 18 | 18 | 19 |
|  | Indigestion, diarrhoea | 24 | 28 | 20 |
